# Effect of *Nigella sativa* on dexamethasone-induced testicular toxicity in mice

**DOI:** 10.22038/AJP.2023.23584

**Published:** 2024

**Authors:** Morteza Abouzaripour, Erfan Daneshi, Saeid Miri

**Affiliations:** 1 *Cellular and Molecular Research Center, Research Institute for Health Development, Kurdistan University of Medical Sciences, Sanandaj, Iran*

**Keywords:** Nigella Sativa, Glucocorticoids, permatogenesis

## Abstract

**Objective:**

The aim of the current study was to assess the effects of Nigella* sativa* essential oil on testicular toxicity in mice induced by dexamethasone.

**Materials and Methods:**

Forty NMRI mice were randomly divided into four groups. The first group (Sham) received 1 ml per day of normal saline by intraperitoneal (i.p.) injection for 7 days. The second group (Control) received (i.p) injection of 5 mg/kg dexamethasone for 7 days. The third group (Dexa+ N.S 5 mg/kg) received dexamethasone (5 mg/kg) and gavaged 5 mg/kg *N. sativa *essential oil for 7 days. The fourth group (N.S 5 mg/kg) for 7 days was gavaged 5 mg/kg *N. sativa*. Histopathology of testis, spermatogenesis, and sperm fertility rate were assessed.

**Results:**

The results of histopathology assessments showed that in the third group, all histopathology criteria were decreased compared to the second group. The number of seminiferous tubules that had abnormal spermatogenesis in Johnsen’s score was slightly decreased in the third group compared to the second group. Furthermore, in the third group, embryo formation criteria were increased.

**Conclusion:**

The data of this research demonstrate that *N. sativa* improves spermatogenesis defects and sperm fertility in mice treated with dexamethasone.

## Introduction

Gluconeogenesis plays pivotal roles in the regulation of biological processes. In fact, glucocorticoids (GC) are required for homeostasis and regulation of a number of physiological events (Oppong and Cato, 2015). The phenomenon of apoptosis caused by GC is very important in biological and clinical terms (Jia et al., 2015). Studies reported that an increase in GC concentration precedes a decrease in testosterone level in the male (Ajdžanović et al., 2017). Testosterone is critical for survival and function of the germ cells in seminiferous tubules (Kavaklı et al., 2011). GCs are anti-inflammatory and immunosuppressive agents (Koźmiński et al., 2020). Dexamethasone is a steroid with catabolic activity that belongs to the GC drugs (Coutinho and Chapman, 2011). Dexamethasone has an effect on testis hemostasis through reduction of testosterone level (Orr and Mann, 1992). Spermatogenesis defects are induced by dexamethasone. Despite this adverse effect, this drug is used in clinic; so, it is necessary to seek for a complement neutralizing its adverse effects (Khorsandi et al., 2013).

*Nigella sativa* (NS) is a herbal plant of the Ranunculaceae family, with several therapeutic effects and multiple beneficial functions in traditional medicine (Ardiana et al., 2020). NS contains many effective components, such as alkaloids (nigelledine and nigellicines), thymoquinone (TQ), flavonoids, saponins (alphahederin), fatty acids, and proteins, that are effective in the treatment of different disorders (Shafiq et al., 2014). *Nigella sativa* has pharmacological effects such as antimicrobial, antioxidant, immune-modulator, anti-inflammatory, anti-arthritic, analgesic, anti-asthmatic, anti-diabetic and anti-carcinogen activities  (Gholamnezhad et al., 2016).

The goal of the current research was to determine possible protective roles of *N. sativa* on toxic effects induced by dexamethasone on spermatogenesis and *in vitro* fertilization. 

## Materials and Methods

### Preparation of Nigella sativa essential oil

In order to extract *N. sativa* essential oil, 100 g of the seeds was ground and immediately distilled by water distillation using a Clevenger apparatus**.** Anhydrous sodium sulfate was used to take the moisture content of the essential oil samples. The accumulated essential oil was placed in dark glass with a tight and glued lid and kept at 4°C.

### Animals and procedure

In the current experimental study, 40 Naval Medical Research Institute (NMRI) mice (42-56 days, 25-30 g) were used. The mice were kept under laboratory conditions (12 hr dark and 12 h light cycle, 50±2 percent of humidity and 22±2°C). The mice were randomly divided into four groups (n=10). The first group (Sham) received 1 ml normal saline intraperitoneal (i.p) injection for 7 days. The second group (Control) received (i.p) injection of 5 mg/kg dexamethasone for 7 days (Chrysis et al., 2003) and received 1 ml normal saline intraperitoneal (i.p) injection for 7 days. The third group (Dexa+ N.S 5mg/kg) received dexamethasone (5 mg/kg) and gavaged *N. sativa* 5 mg/kg for 7 days. The fourth group (N.S 5mg/kg) gavaged N*. sativa* 5 mg/kg for 7 days (Ait Mbarek et al., 2007). The study protocol obtained approval from the Kurdistan University of Medical Sciences (Code: IR.MUK.REC.1395/293).

### Histopathology

One day after the last injection, animals were sacrificed by cervical dislocation under anesthesia (ketamine 95% an xylazine 5%) for further experiments. The right testis was fixed in Bouin’s fluid from each animal and then embedded in paraffin. Sections with a thickness of 5 μm stained with haematoxylin and Eosin (H&E) for histopathology assessment and Johnsen’s scoring.

From each mouse, 6 slices and for each slice, 3 fields were obtained. Slices were evaluated for detachment (spermatocyte sequestration from epithelium of seminiferous tubules), sloughing (cell mass from germinal cells into lumen) and existence of vacuoles within the seminiferous tubules. For each treatment, the average percentage of normal and regressed tubules was determined. For each sample, the number of tubules containing vacuoles, sequestration and sloughing in each field was divided by the whole number of tubules in that field then, the number was multiplied by 100. 

### Spermatogenesis

Germinal epithelium was graded by using the modified Johnsen’s scoring method. In each field, the seminiferous tubules were assessed under magnifications of 40 x. Inactive tubules received grade 1, and tubules containing at least 5 spermatozoa received grade 10 (Johnsen 1970).

### In vitro fertilization

Sperms of 8-week old male mice from were removed the tail of epididymis. Suspension of sperm (1×10^6^ motile spermatozoa/ml) was capacitated (1 h in 500 μl human tubular fluid (HTF) culture medium for 10 min at 37ºC). After cell detachment, a concentration of 1×10^6 ^ml was placed in an incubator for 1 h for capacitation. Matured oocytes were added to 100 μl droplets of HTF that contained 0.1 ml of capacitated spermatozoa. After incubation (5 h), the oocytes were washed with HTF medium and checked for the presence of second polar body and formation of pronuclei indicating fertilization. Oocytes were cultured in M2 medium and assessed for cleavage to the two cells stage after 1 day.

### Statistical analysis

Results were analyzed by using the SPSS 22. Data were analyzed using One-way ANOVA with a U Mann-Whitney and are presented as mean±SD. A p<0.05 was considered statistically significant.

## Results

### Histopathology

**Figure 1:**
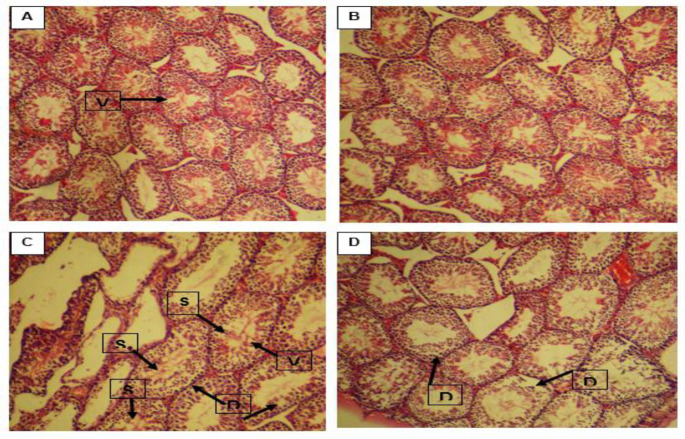
Light microscopy of cross sections of H&E-stained testis from the study groups. A: Group 1, B: Group 4, C: Group 2, and D: Group 3. V: vacuole, S: slough, and D: detached. magnification ×100.

Sections of the testis in the first group showed normal spermatogenesis with a low occurrence of sloughed, vacuolized or detached seminiferous (Figure 1a). Normal structures of the seminiferous and entire germinal epithelium in the fourth group were observed (Figure 1b). There was no difference between the forth and first groups in the histopathology criteria (p>0.05). Seminiferous atrophy was significantly higher in the second group than in the first group (p<0.01, Figure 1c). In the third group (Figure 1d), all histopathological criteria were decreased compared to the second group (p<0.05). The data of histopathology evaluation are shown in Table1.

### Spermatogenesis 

**Figure 2:**
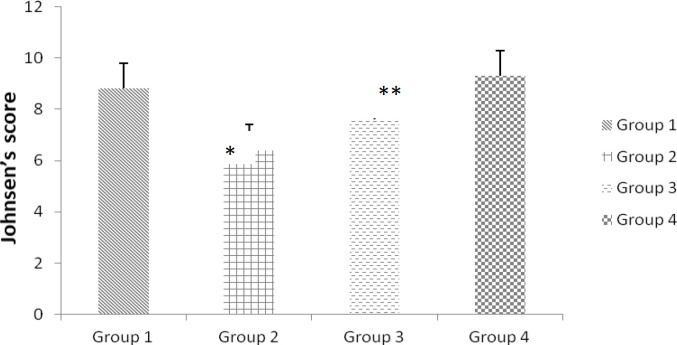
Johnsen’s score in the four groups. Values are expressed as mean±SD for 24 mice. *Compared to group 2 and 1. **Compared to group 3 and 2

By using the Johnsen’s scoring method maturity of the germinal epithelium was graded. In the first and forth groups, normal spermatogenesis was observed and there were no significant differences between the two groups. Incomplete spermatogenesis was found in the second group and the mean Johnsen’s score was less than the first group (p<0.05). In the third group, few tubules had abnormal spermatogenesis and the mean Johnsen’s score was slightly increased compared to the second group (p<0.01). Johnsen’s scoring result is shown in Figure 2.

### In vitro fertilization (IVF)

**Figure 3:**
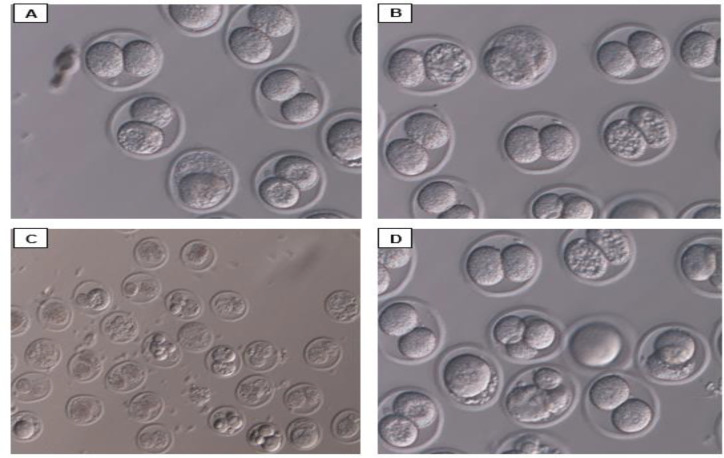
Photomicrography of embryo. A: group 1 C: Group 2. D: Group 3. B: group 4

Table 2 shows that the forth group had a higher rate of embryo formation compared to the first group although there were no differences between the two groups*.* In the second group, formation of embryo was lower than the first group (p<0.05). In the third group, embryo formation criteria were increased compared to the second group (p<0.01, Table 2 and Figure 3).

## Discussion

We found that dexamethasone could disrupt spermatogenesis, and fertility potential. Dexamethasone also increased cell sloughing from the germinal epithelium and created vacuoles in the seminiferous tubules. These changes were abrogated using essential oil of *N. sativa*.

Histopathologic studies showed that dexamethasone induced generation of vacuoles in different sizes in germinal epithelium, and in some parts, it caused tubular atrophy. Vacuole formation in epithelium of seminiferous tubules was also induced using toxic agents, hypophysectomy, transient exposure to high temperature and androgen exposure (Hooley et al., 2009). Slough is produced by the effects of chemical substances on Sertoli cells (Jeffrey et al., 2007). These effects could cause sequestration in germ cell division and an increase in the rate of abnormal spermatids (Hess and Nakai, 2000). In the current study, dexamethasone caused a decrease in the Johnsen test score, indicating its adverse effects on spermatogenesis. Hashemitabar and colleagues attested a noticeable increase in the rate of apoptosis in germinal sperm cells after exposure to 7 and 10 mg/kg dexamethasone (Hashemitabar et al., 2008). Glucocorticoids cause a defect in the immune system, so the cells under exposure to them would be more sensitive to environmental stressors, thereby increasing the rate for apoptosis in the cells. From what has been mentioned, it could be speculated that the decrease in the number of germinal sperm cells in seminiferous tubules is probably due to a reduction in the proliferation and/or an increase in the apoptosis of germinal sperm cells. Long-term dexamethasone administration could affect spermatogenesis and hypophyseal-testis hormonal axis resulting in a retardation of testis function (Orazizadeh et al., 2010). Hashemitabar and colleagues in another study examined the role of dexamethasone on pro-apoptotic Bax expression in mouse stem cells, and they noticed a positive link between dexamethasone administration and Bax expression (Hashemitabar et al., 2008). *Nigella sativa* increased testosterone, Gonadotropin-releasing hormone (GnRH), and antioxidant enzymes and decreased the levels of free radicals in rats treated with chlorpyrifos (Rachid et al., 2014). Also another study showed an improvement in sperm motility and vitality following *N. sativa* administration (Abouzaripour et al., 2019). Al-Sa'aidi and colleagues found that *N. sativa* could increase the size and thickness of seminiferous tubules, improve spermatogenesis, and increase the number of spermatogonia, primary spermatocytes, secondary spermatocytes, spermatids and sperms (Al-Sa'aidi et al., 2008). In the current study, the number of seminiferous tubules that had abnormal spermatogenesis in Johnsen’s score was slightly decreased in animals treated with *N. sativa*.

In this study, we showed that *N. sativa* could improve fertility in mice receiving dexamethasone. In the study carried out by Kamarzaman and colleagues, the results revealed that thymoquinone could protect mice affected by cyclophosphamide. It also improved fertility potential and reduced the number of defective blastomeres, and, therefore, they introduced thymoquinone as an anti-oxidant for preservation of embryos (Kamarzaman et al., 2014).

*N. sativa* has a healing effect against dexamethasone-induced testicular cell damage. Due to financial and time constraints, apoptotic genes were not studied. Examination of these genes in future studies could provide new information. 

**Table 1:** Testis histopathology assessments

			**Percentage tubules**	**Groups**
**Vacuolized**	** Sloughed**	**Detached**	**Normal**	
4.51±0.94 * 19.11±1.89 **10.88±1.17 0.94±4.48	3.8±0.69 0.56±8.08* 0.56±6.1** 0.70±3.2	9.6±0.84 *26.47±0.84 **22.44±1.76 8.3±0.93	81.93±0.82 *46.32 ±0.94 **60.54±1.91 83.97±0.73	**Group 1** **Group 2** **Group 3** **Group 4**

Values are expressed as mean±SD. *Compared to group 2 and 1. **Compared to group 3 and 2

**Table 2:** The number of oocytes attaining the embryo formation and destruction

**Number of two-cells stage (%)**	** Number of oocyte**	
31.00±0.4 0.03±34.00 0.08±8.00* 0.05±**12.00	105 115 75 90	**Group 1** **Group 4** **Group 2** **Group 3**

Values are expressed as mean±SD. *Compared to group 2 and 1. **Compared to group 3 and 2.
